# Case of Supposed Labour, in Which a Mass of Hydatids and a Placenta Were Brought Away, without Any Fœtus Being Discovered; with Some Observations on Retroversion of the Uterus

**Published:** 1815-05

**Authors:** John Charles Collins

**Affiliations:** one of the Physicians to the Swansea Dispensary.


*For the Medical and Physical Journal.*

### *Case of supposed Labour, in which a Mass of Hydatids and a Placenta were brought away, without any Fœtus being discovered; with some Observations on Retroversion of the Uterus;* by JOHN CHARLES COLLINS, M.D. F.L.S. one of the Physicians to the Swansea Dispensary.

FEBRUARY 23, 1815, I was sent for to see Mrs. J. supposed to be in labour by the midwife who usually attended her, who informed me she had been lingering the last thirty hours; and that, just before they sent, she had, during a slight pain, passed a something, supposed to be the placenta, and which, upon my examining, I found to be a congeries of hydatids, forming a globular mass, of the size of a large lemon. But, as a considerable discharge continued, I immediately examined and found the os tincæ much dilated, and a soft mass protruding and filling it up. I remarked it had an unusual cold sensation. A slight pain soon coming on, I brought it away, followed by a copious discharge. This appeared to be a substance of a similar kind to the first; and, upon placing my hand on the abdomen, I found the uterus contracted to the usual size it is felt after regular parturition. Ordering her an opiate, I left the room, to examine more particularly the two substances which had been produced. The first appeared a complete mass or congeries of hydatids, of the size of small nutmegs, united together on all sides; upon cutting through which, I could not find any remains of a fœtus; the centre composed of a number of connected blood-vessels, and I discovered what I supposed to be the umbilical cord, ruptured close to its entrance into the mass. The hydatids were of a very yellow colour; and the whole appearance of the substance reminded me of a very closely connected bunch of grapes. The second portion was evidently the placenta, about the size of a small full-timed one, one surface thickly covered with pendulous hydatids, of various sizes, from that of a large olive to a lupin seed. The necks of the larger were more than an inch long, with many smaller ones. The uterine vessels of the placenta evident on one side, and the remaining portion of the funis very small, and about four inches long. I inquired of my patient the history of her case, and she stated as follows:—That she is 27 years old; has had five living children; and is of a delicate constitution. She supposed herself in her seventh month of pregnancy; speaks confident as to her having felt the child at the fourth month, the usual time she quickens; that it was about the fifth month month a change took place; her breasts became flaccid; her size diminished; and she always dreaded that the child died at that time, though she could not attribute it to any accident. Three days before I saw her, from a little over exertion, she brought on a coloured discharge, which continued until about thirty hours before I saw her, when regular but slight pains came on, and during one of which the first portion came off, when they were alarmed, and sent for me. Her general health, from the fifth month, declined fast. The usual discharge continued after the passage of the second mass, in the same manner as if she had been regularly confined, and she is now completely recovered.

Cases of false conception, mola, and hydatids, are frequently to be met with in medical authors; but I do not recollect reading of any case exactly similar to this, for I look upon the child having lived to quicken, or become of that size that it arose out of the pelvis, as almost certain; there was no reason for deception. Smellie* supposes that, should the child die in the first or second month, it may be entirely dissolved, and even at a later period. Denman† defines such cases ovum deforme, and arranges them under the class of monsters; but adds, "if carefully examined with a knife, various parts of a child may be discovered." The change here was so complete as not to leave a vestige behind. Burns‡ says, "moles are generally expelled within three months, or usual time of quickening; but adds, no motion is felt, or child can be perceived, and the breasts are flaccid. In Mrs. J.'s case, motion was felt, and the breasts did not become flaccid, but were enlarged, as usual, until after that period; but he quotes a case where the lochia followed expelled hydatids. In Home's§ case, which terminated fatally, the hydatids were attached to the surface of the membrane, and he supposed it a general affection of the amnion; and Denman supposed them sometimes an original production of the uterus. Cases wherein the placenta is affected, or generates hydatids, may be numerously quoted; but in Mrs. J.'s case there appeared a considerable difference in shape and colour, between those attached to the placenta, and those forming the fœtal mass. Those attached to the former corresponded to the description given by Dr. Bailey,|| consist-

* Smellie, vol. i. p. 128.

† Denman, vol. i. p. 118.

‡ Burns's Midwifery, p. 79.

§ Transact. of Med. and Chirurg. Society, vol. ii. p. 300.

|| Bailey's Morbid Anatomy, p. 378.

 ing ing of vesicles of an oval shape, with a narrow stalk or neck to each. Home,* in his Croonian Lecture, Nov. 11, 1790, mentions two species, one globular, another of an oval form. Dr. Monro† enumerates seven heads found in the human body: his sixth species, he says, is uncommon, being laterally united to each other. As in the case of the first portion expelled, his seventh species are those generally attached to the placenta. Dr. Parr states the number of varieties as eighteen, but only describes ten. The above case bears some analogy to one related by Mr. Lemon:‡ in both a placenta was found, but no foetus. Mrs. J.'s case is more remarkable, from her positive conviction that she felt the child at the usual time. Was it possible that during the following three months the foetus could have been so completely destroyed as to leave no vestige behind? Smellie states that a child of three months may be so dissolved in fourteen or fifteen days. The difference in the form of the hydatids on the fœtal and placenta masses, was also remarkable. In a judicial point of view, these cases are particularly interesting, as they prove the existence of a placenta without a fœtus, or the fœtus so changed as not to be recognized; and show how particularly cautious medical men should be in giving their evidence in cases of suspected delivery, where they have not an opportunity of examining what has been discharged. Cases of this kind excite a peculiar interest in my mind, from the circumstance of my being extensively engaged in the female department of medical practice, during which I have often met with cases of mola, false conceptions, and hydatids, as well as my holding the situation of coroner in an extensive and populous district in this county; and I do not hesitate at declaring it as my opinion, that the mark of a placenta on the uterus is not an indubitable proof of the existence of a child, though I think it is so of sexual intercourse having taken place.

*Retroversion of the Uterus.*—Since the public demonstration, by Dr. Hunter, of a case of retroverted uterus, in 1754, the subject has engaged the attention of many eminent men, and the symptoms and deranged position been clearly explained, though still a difference of opinion exists as to the cause, whether it is owing to the distension of the bladder, or relaxation of the parts. During my extensive practice in

* Med. Inst. and Obs. vol. viii. p. 193.

† Monro's Morbid Anat. p. 257.

‡ Edinburgh Journal, January 1815.

midwifery midwifery for the last fifteen years, nine cases of retroversion have occurred to me; and, I am happy to say, all have terminated well. About ten years since, the two first happened; and, at that time, I was induced to think it absolutely necessary, after the extraction of the urine, to make use of those means recommended for the purpose of replacing the uterus, yet a recurrence of the displacement frequently took place upon the most trifling exertion, until the growth of the fœtus had sufficiently increased in size to prevent it. Under these circumstances, and being convinced that relaxation of the parts was a very frequent cause of the repeated fall of the fundus, frequently and greatly promoted by a distended bladder, I was induced, in the subsequent cases, to direct cold vinegar and water to be applied to the parts immediately upon the water being drawn off; to be repeated three or four times daily, particularly after each time of passing urine. By these means, my patients have been enabled to take sufficient exercise, for the purposes of general health. In three cases, I have been under the necessity of directing a piece of sponge, filled with vinegar and water, to be introduced, for ten minutes or a quarter of an hour, three or four times a-day; and, excepting in these three cases, I have never been under the necessity of introducing the catheter more than once; and thus the very unpleasant repetition of drawing off the urine, which, to a delicate female mind, is always irritating, has been obviated, exclusive of the very inconvenient absence of my patients from domestic duties being prevented; for, as far as my experience goes, it corroborates an observation of Dr. Denman as to the particular class of society most liable to be affected. He says, "I shall conclude these remarks (which, by the by, are excellent) with an observation which will appear extraordinary. Women who live in an humble situation of life, or in an unrefined state of society, are scarcely ever liable to this complaint, because they are free from the constraint of company; and those in the highest rank of the most refined society, not being abashed to withdraw from company, are nearly in the same situation; but those who are in a middle state of life, with decent, yet not over-refined, manners, have not cast off the bashfulness of the former, nor acquired the freedom of the latter, are most subject to the retroversion of the uterus."

JOHN CHARLES COLLINS.

